# Radiology report format preferred by requesting physicians: prospective analysis in a population of physicians at a university hospital

**DOI:** 10.1590/0100-3984.2018.0026

**Published:** 2019

**Authors:** Denise Maria Rissato Camilo, Tiago Kojun Tibana, Isa Félix Adôrno, Rômulo Florêncio Tristão Santos, Camila Klaesener, Walberth Gutierrez Junior, Edson Marchiori, Thiago Franchi Nunes

**Affiliations:** 1 Hospital Universitário Maria Aparecida Pedrossian da Universidade Federal de Mato Grosso do Sul (HUMAP-UFMS), Campo Grande, MS, Brazil.; 2 Universidade para o Desenvolvimento do Estado e da Região do Pantanal (Uniderp), Campo Grande, MS, Brazil.; 3 Universidade Federal do Rio de Janeiro (UFRJ), Rio de Janeiro, RJ, Brazil.

**Keywords:** Radiology information systems, Medical records, Referral and consultation, Tomography, X-ray computed, Ultrasonography, Sistemas de informação em radiologia, Registros médicos, Encaminhamento e consulta, Tomografia computadorizada, Ultrassonografia

## Abstract

**Objective:**

To improve communication between attending physicians and radiologists by defining which information should be included in radiology reports and which reporting format is preferred by requesting physicians at a university hospital.

**Materials and Methods:**

Respondents were asked to choose among reports with different formats and levels of detail, related to three hypothetical cases, and questioned as to which characteristics commonly found in radiology reports are appropriate for inclusion. To assign the absolute order of preference of the different reports, the Kemeny-Young method was used.

**Results:**

Ninety-nine physicians completed the questionnaires (40.4% were resident physicians; 31.3% were preceptors of residency programs; and 28.3% were professors of medicine). For ultrasound with normal findings, ultrasound showing alterations, and computed tomography, respectively, 54%, 59%, and 53% of the respondents chose structured reports with an impression or comment. According to the respondents, the characteristics that should be included in the radiology report are the quality of the image, details of the clinical presentation, diagnostic impression, examination technique, and information about contrast administration, selected by 92%, 91%, 89%, 72%, and 68%, respectively. Other characteristics that were considered important were recommendations on follow-up and additional radiological or non-radiological investigation.

**Conclusion:**

Requesting physicians apparently prefer structured reports with a radiologist impression or comment. Information such as the quality of the examination, the contrast agent used, and suggestions regarding follow-up and additional investigation are valued.

## Abstract (pt)

**Objetivo:**

Melhorar a comunicação entre médicos assistentes e radiologistas, definindo quais informações deveriam ser incluídas num relatório radiológico e qual o formato preferido dos solicitantes de um hospital universitário.

**Materiais e Métodos:**

Os participantes foram convidados a escolher a opção preferida entre relatórios com diferentes formatos e níveis de detalhamento de três casos hipotéticos e questionados se características comumente encontradas em um laudo radiológico são apropriadas ou não para inclusão. Para atribuição da ordem absoluta de preferência dos diferentes relatórios, o método Kemeny-Young foi utilizado.

**Resultados:**

Noventa e nove médicos responderam os questionários (40,4% médicos residentes, 31,3% preceptores dos programas de residência e 28,3% professores da faculdade de medicina). Laudos estruturados com impressão ou comentário foram selecionados nas situações de ultrassonografia normal por 54% dos participantes, ultrassonografia alterada por 59% e tomografia computadorizada por 53%. As informações que deveriam ser incluídas, de acordo com os solicitantes, foram qualidade da imagem (92%), detalhes do cenário clínico (91%), impressão diagnóstica (89%), técnica do exame (72%), informações sobre meio de contraste (68%). Opiniões sobre seguimento e investigação radiológica ou não radiológica se mostraram importantes.

**Conclusão:**

Relatórios estruturados com impressão ou comentário de um radiologista são prepostos. Informações como qualidade de exame, meio de contraste utilizado e sugestões de acompanhamento e investigação adicional são valorizadas pelos médicos solicitantes.

## INTRODUCTION

The radiology report is vital for patient management. Radiologists play an important role in patient care through the accurate interpretation of imaging studies and appropriate communication of the imaging findings to attending physicians. Although some attending physicians can interpret imaging studies on their own, a report prepared by a radiologists has proven to be a more accurate and comprehensive way of interpreting the findings, resulting in better patient care^(^^1^^-^^5^^)^.

To improve patient care, it is imperative that radiology reports be timely and accurate, as well as that they primarily address the clinical issue in question. For a health care system, these may be the most important and easily available metrics to quantify the value of the radiology services being provided. Although learning to create an imaging report is an essential component of residency programs in radiology and diagnostic imaging, less than 1 hour per year is devoted to formal training on how to frame a radiology report^(^^6^^)^. Instead, most trainees and residents learn the art of reporting by observing professors, senior residents, and fellow students.

Traditionally, radiology reports employed free-text, narrative language. Studies have shown that the use of unstructured reports written in narrative language could be an obstacle to optimal patient care. Excessive variation in language, length, or style can reduce the clarity of the report, making it difficult for physicians to identify the key information needed for patient care^(^^7^^-^^10^^)^.

The use of a structured format has been advocated as a potential means of improving the quality of radiology reports. Increasingly, medical schools have taught the use of the structured forms in radiology. Therefore, the main objective of this study was to improve communication between radiologists and attending physicians, defining which format is preferred by attending physicians at a university hospital. The secondary objective was to evaluate the level of acceptance of structured reports for ultrasound and computed tomography (CT).

## MATERIALS AND METHODS

Between December 2017 to February 2018, an electronic questionnaire was sent to 260 doctors at a university hospital. We received a total of 99 completed questionnaires: 85 were received through a software application (Survey Monkey via WhatsApp); and 14 were delivered in person. All of the respondents belonged to one of three groups: medical school professors, preceptors of residency programs, and resident physicians.

The main body of the questionnaire was divided into three sections. In the first section (Table 1), there were items related to the respondent category (professor, preceptor, or resident), specialty (clinical, surgical, pediatrics, obstetrics/gynecology, orthopedics, pathology, or diagnostic imaging), and time since graduation. The respondents were also asked to specify how many imaging reports they received each week. In the second section (Table 2), respondents were asked to select which common features are appropriate for inclusion in a radiology report by answering yes or no for each item. In the third and final section (Appendix), several reports, with different formats and levels of detail, were provided. Respondents were asked to rank them by order of preference.

**Table 1:** Questionnaire to determine the profile of the respondents.

Profile of the interviewee within the context of the Federal University of
Mato Grosso do Sul (UFMS):
- Professor of Medical School - UFMS
- Preceptor of a residency program at the University Hospital - UFMS
- Resident physician
Specialty:
Time since graduation:
< 5 years 5-10 years 10-15 years 15-20 years > 20 years
Number of radiology (CT or ultrasound) reports reviewed per week:
0-10 10-20 20-30 30-40 > 40

**Table 2:** Questionnaire related to the items to be included in radiology reports.

Do you think the following items should be included in the body of the radiology (CT or ultrasound) report?
1 - Indication for the clinical examination/complaint: Yes/No Example: Jaundice two months prior
2 - Examination technique: Yes/No Example: Volumetric acquisition with thin slices and planar reconstructions
3 - Name, dose, concentration, route of administration and infusion rate
of the contrast medium used: Yes/No Example: Iobitridol 350 mg/mL 60 mL intravenous 4,5 mL/s
4 - Image quality: Yes/No Example: Respiratory movement artifacts impede the evaluation of the image
5 - Dimensions of normal organs: Yes/No Example: The right lobe of the liver measures 14 cm on the longitudinal axis
6 - Recommendation for serial imaging (follow-up): Yes/No Example: We suggest a follow-up tomography, with a low dose of radiation, in 3 months
7 - Recommendation for pathological analysis (biopsy): Yes/No Example: We suggest histological analysis of the aforementioned lesion for better diagnostic definition
8 - Recommendation for further investigation with another imaging examination: Yes/No Example: Magnetic resonance imaging may provide additional information
9 - Recommendation for further investigation with a non-radiological examination: Yes/No Example: We suggest further investigation with colonoscopy
10 - Conclusion (diagnostic impression): Yes/No Example: Taken together, the findings suggest pancreatic neoplasia
11 - Bibliographic reference at the end of the report: Yes/No

We selected two hypothetical scenarios for ultrasound examinations of the upper abdomen and one hypothetical scenario for abdominal CT. For the ultrasound reports, the first scenario was the case of a patient with a history of weight loss (15 kg in two months), in which there was clinical suspicion of malignancy. The second scenario was the case of a patient with right upper quadrant abdominal pain, in which there was clinical suspicion of a gallstone. The structures of the ultrasound reports were similar. For the CT report, the scenario was the case of a patient with a two-day history of pain in the right iliac fossa, in which there was clinical suspicion of acute appendicitis. For each scenario, the were four reporting options, all of which were identical in terms of content. Whereas the first two reports were very basic, the third consisted of a more detailed report, including a diagnostic impression, and the fourth was a structured report modeled after that proposed by the American College of Radiology^(^^11^^)^. To assign the absolute order of preference for the different reports, the Kemeny-Young method was used^(^^6^^,^^7^^)^.

## RESULTS

### Profile of the respondents

Of the 99 completed questionnaires, 40 (40.4%) were obtained from resident physicians, 31 (31.3%) were obtained from preceptors of residency programs, and 28 (28.3%) were obtained from medical school professors. A wide variety of specialties were represented, including internal medicine (37.4%), general surgery (28.3%), obstetrics/gynecology (11.1%), diagnostic imaging (9.1%), pediatrics (1.0%), orthopedics (5.0%), and pathology (2.0%). Of the 99 respondents, 24 (24.3%) receive ≤ 10 radiology reports per week, 26 (26.3%) receive 10-20 per week, 22 (22.1%) receive 20-30 per week, 9 (9.1%) receive 30-40 per week, and 18 (18.2%) receive > 40 per week. The time since graduation was < 5 years in 26.3% of the respondents, 5-10 years in 29.2%, 10-15 years in 13.1%, 15-20 years in 9.1%, and > 20 years in 23.2%.

### Characteristics of the reports

Table 3 shows the respondent opinions regarding which of the components typically included in a radiology report should in fact be included. Preference was given to details related to the clinical context, the examination technique, and the quality of the images; details related to the use of contrast media; recommendations for additional imaging examinations or follow-up, as well as recommendations for additional investigation with non-radiological methods; and the diagnostic impression. Opinions were equally split regarding the inclusion of bibliographic references in special situations.

**Table 3:** Respondent opinions regarding the components of a radiology report.

Should the following information be included?	Yes	No
Details of the clinical context	91%	9%
Examination technique	74%	26%
Contrast medium administered	68%	32%
Image quality	92%	8%
Imaging follow-up	89%	11%
Recommendation for additional pathological analysis	78%	22%
Recommendation for additional imaging examinations	84%	16%
Recommendation for additional non-radiological investigation	79%	21%
Diagnostic impression	89%	11%
Bibliographic references	55%	45%

### Preference by type of report

#### Report for an ultrasound examination showing alterations

**Figure 1:**
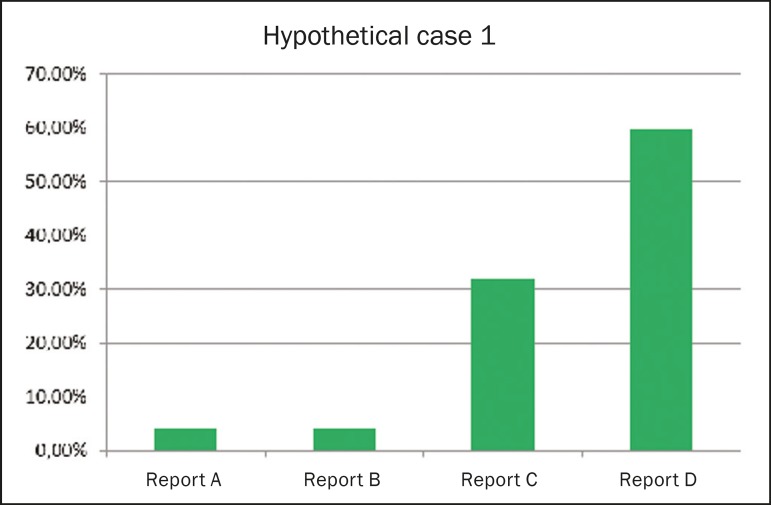
Frequency of each style of reporting ranked as the best option for an ultrasound report showing alterations.

Figure 1 shows the frequency of each style of reporting ranked as the best option for an ultrasound report showing alterations. Report D was preferred by 59.8% of the respondents. The order of preference of ultrasound reporting styles (from most preferred to least preferred, as determined by the Kemeny-Young method) was D-C-A/B-A and B each being preferred by 4.1% of the respondents.

#### Report for an ultrasound examination showing no alterations

**Figure 2:**
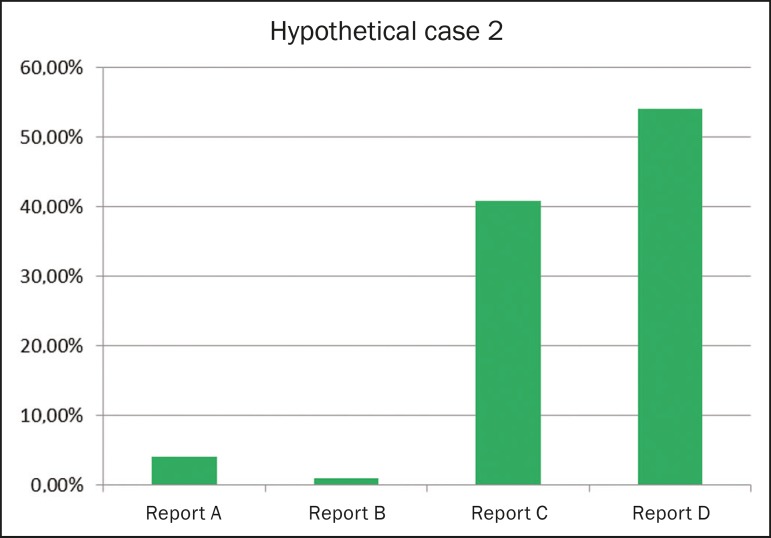
Frequency of each style of reporting ranked as the best option for an ultrasound report showing no alterations (i.e., with normal findings).

Figure 2 shows the frequency of each style of reporting ranked as the best option for an ultrasound report showing no alterations (i.e., with normal findings). Report D (a structured, detailed report with diagnostic impression) was preferred by 54.2% of the respondents. The order of preference of ultrasound reporting styles (from most preferred to least preferred, as determined by the Kemeny-Young method) was D-C-A-B.

#### Report for a CT examination

**Figure 3:**
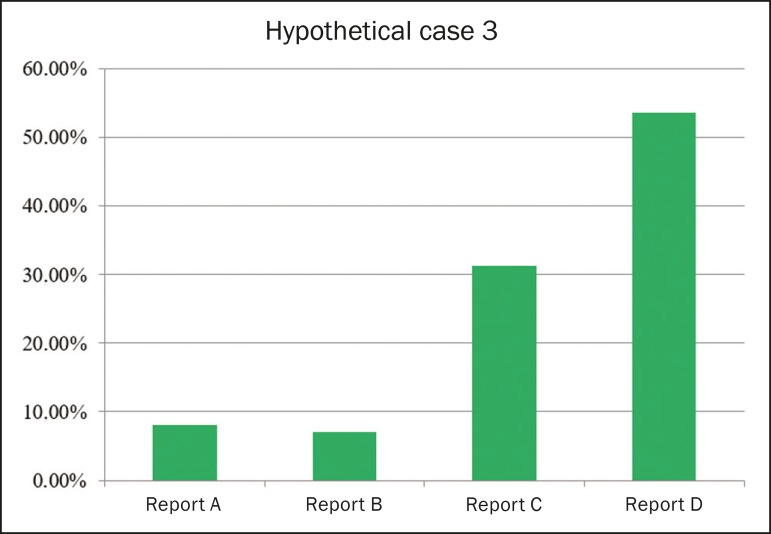
Frequency of each style of reporting ranked as the best option for a CT report.

Figure 3 shows the frequency of each style of reporting ranked as the best option for a CT report. The order of preference of CT reporting styles (from most preferred to least preferred, as determined by the Kemeny-Young method) was D-C-A-B, each being preferred by 53.5%, 31.3%, 8.1%, and 7.1% of the respondents, respectively.

## DISCUSSION

There is as yet no consensus regarding the amount of information that an imaging report should contain or regarding what is the best report format from the point of view of the requesting physicians. Highly detailed reports are extremely valuable for good patient care, to serve as a forensic record, and to assist in the retrospective interpretation of images, as well as their interpretation in multidisciplinary team meetings. However, superfluous information can distract from the main message of a radiology report and prevent the requesting physician from understanding which are the main findings of a specific examination. It is crucial that the report be structured in a way that maximizes and optimizes the transfer of information to the attending physician. Determining the preferences of the recipients of such reports is quite relevant, in that it can promote effective communication between radiologists and requesting physicians.

In our study, some of the details related to the examination technique and image quality were considered appropriate for inclusion in the body of the radiology report. Likewise, the recommendation for additional examinations was considered an integral component, as was the diagnostic impression.

Previous studies have shown that radiologist recommendations regarding follow-up, treatment, or referral are considered unnecessary^(^^12^^)^, suggesting that the inclusion of such data could be left to the discretion of the radiologist. In such analyses, when an additional examination was recommended by the radiologist in the report, more than one third of requesting physicians preferred that they be the ones to order additional examinations (i.e., that no additional measures be suggested in the report). The reason given was that radiologists might not be aware of the clinical status of the patient and that the indication might therefore be inappropriate at that time^(^^12^^)^.

Our analysis showed that the recommendations for follow-up and ongoing investigation were considered particularly necessary, underscoring the important role that radiologists play in the management of patients. We find that interesting in view of the seemingly growing expectation that radiologists will go beyond the performance of examinations and provision of reports, taking responsibility for a greater proportion of the clinical problems presented by patients^(^^13^^)^.

We observed a trend toward a preference for radiology reports that are more detailed over simpler reports. The diagnostic impression also proved to be a highly valued feature in a report. Although the numbers of respondents in each individual specialty were small in our sample, there were no differences between the specialties in terms of respondent preference for details. The preference for detailed reports is in accordance with the findings of previous studies^(^^9^^,^^14^^)^. However, it has been observed that preferences change depending on the clinical context^(^^14^^)^.In the present study, a small number of respondents stated that multidisciplinary discussions with radiologists in the hospital are more important than are well-structured reports, especially in cases of emergency.

Establishing a reporting base that allows the generation of structured reports with comments from the radiologist is crucially dependent on the adaptability of the health care facility, as well as on the capacity and desire to use such reports. Radiologists who remain up to date are increasingly computer literate, a trend aided by the dissemination of the PACS, which makes the use of structured reports more palpable today^(^^12^^)^, given that there is a possibility of storing report templates, according to the preference of the service, composing a database with predetermined models of structured reports.

A recurring theme in this and other studies is the suggestion that requesting physicians are often unclear on the clinical data, when any specific organ has been examined and considered normal, or has not been examined, a problem that can be circumvented through the use of structured reports^(^^9^^,^^12^^,^^14^^)^. We believe that the use of structured reports would lead to a significant increase in the time required to issue such reports and, consequently, in the workload. However, the effective use of models and pre-reports at facilities where there are attending physicians and residents, would minimize the negative impacts. Although there may be a slight increase in the amount of time required to produce each report, the advantages of more consistent reporting and fewer confounding factors for requesting physicians should be considered. In addition, structured reports, if properly stored, could generate a significant resource for future research in PACS system knowledge bases^(^^12^^)^.

In the present analysis, we included specialists in diagnostic imaging, because they are generally also requesting physicians and receive reports. We also included specialists in interventional radiology, angiography, interventional (hemodynamic) cardiology, vascular/endovascular surgery, and echocardiography.

The limitations of this study include the relatively small number of medical professionals, the fact that all of the respondents were recruited from a single center, and the fact that we focused on only two methods (ultrasound and CT), excluding other imaging methods. Although the study was conducted at a public university hospital, the vast majority of the respondents also work in private networks, suggesting that our results could be extrapolated to such facilities. We believe that the use of a more comprehensive questionnaire, addressing the other imaging methods typically used in clinical practice, such as magnetic resonance imaging, conventional radiography, and contrast-enhanced examinations, might generate response rates sufficient to form truly representative results. Multicenter studies involving larger patient samples are needed in order to test our hypothesis.

## CONCLUSION

A structured report presenting a final conclusion or comment has proven to be the style preferred by attending and requesting physicians, whether or not the report describes alterations. Information on examination quality and the contrast medium used were considered important features of a radiology report, as were recommendations for additional tests, as well as a diagnostic impression.

## Appendix

### ULTRASOUND REPORT

**Scenario 1:**

Patient referred for investigation of abdominal pain and weight loss. Suspicion of malignancy. Ultrasound of the upper abdomen requested.

**Final diagnosis:** Liver nodules suspected of being secondary implants or metastases.

REPORT A

Poorly defined hypoechoic nodules in the right lobe of the liver, measuring between 1.1 and 3.8 cm, with a suspicious aspect.

No alterations observed in the pancreas, bile ducts, gallbladder, spleen, kidneys, or abdominal aorta.

REPORT B

**Table:**

Liver	Normal dimensions and morphology.
	Three poorly delimited hypoechoic nodules in the right lobe of the liver, measuring 2.0 x 1.5 x 1.5 cm, 1.1 x 1, 5 x 1.7 cm, and 3.8 x 3.5 x 3.2 cm, respectively.
Pancreas	Normal
Gallbladder	Normal wall thickness; anechoic content without calcifications.
Biliary tract	No dilatation of intrahepatic or extrahepatic bile ducts.
Spleen	Normal
Kidneys	Normal
Abdominal aorta	Normal caliber and trajectory.

REPORT C

Liver of normal dimensions, with regular contours and thin borders. Hepatic parenchyma with preserved echogenicity and echotexture. Three poorly defined hypoechoic nodules observed in the right lobe of the liver, measuring 1.1 x 1.5 x 1.7 cm, 2.0 x 1.5 x 1.5 cm, and 3.8 x 3.5 x 3.2 cm, respectively. Portal vein with normal caliber and trajectory.

Normally distended gallbladder with thin, regular walls. Anechoic vesicular content without calcifications.

Intrahepatic bile ducts without dilatations.

Hepatobiliary duct of normal caliber.

Pancreas of normal size, echotexture, and echogenicity. There is no dilatation of the main pancreatic duct.

Spleen of normal size, echotexture, and echogenicity.

Kidneys with normal dimensions and regular contours. Parenchyma of normal thickness and preserved echogenicity, with good corticomedullary differentiation. There is no evidence of calcifications. No dilation of the renal pelvis or ureter.

Abdominal aorta with normal caliber and regular trajectory.

**Conclusion:** Hepatic nodules of indeterminate nature.

REPORT D

**Liver**

Size: normal

Craniocaudal dimension: 13 cm

Echogenicity: normal

Contours: regular

Presence of poorly delimited hypoechoic nodules (size and location):

- 3.8 x 3.5 x 3.2 cm in segment VI

- 2.0 x 1.5 x 1.5 cm in segment VI

- 1.1 x 1.5 x 1.7 cm, in segment VII

**Biliary tract**

Intrahepatic ducts: normal

Common bile duct diameter: 3 mm

**Gallbladder**

Normal

Calcifications: absent

Bile sludge: absent

Thickening of the gallbladder wall: absent

**Pancreas**

Head and uncinate process: normal

Body and tail: not viewed

**Spleen**

Splenomegaly: absent

Craniocaudal dimension: 10 cm

**Right kidney**

Normal

Hydronephrosis: absent

Size: 11 cm

**Left kidney**

Normal

Hydronephrosis: absent

Size: 12 cm

**Abdominal aorta and inferior vena cava**

Segments viewed are normal

**Ascites/fluid within the cavity**

Absent

**Conclusion:** Hepatic nodular images of indeterminate nature.

### ULTRASOUND REPORT

**Scenario 2:**

Patient with pain in the right hypochondrium and a suspected gallstone. Ultrasound of the upper abdomen requested.

**Final diagnosis:** Ultrasound findings indicative of a normal abdomen.

### COMPUTED TOMOGRAPHY REPORT

**Scenario 3:**

Patient with pain in the right iliac fossa and with clinical suspicion of appendicitis. Abdominal CT with intravenous contrast requested.

Final diagnosis: Acute appendicitis.

REPORT A

Signs of uncomplicated appendicitis, characterized by an enlarged appendix with wall thickening. Densification of the adjacent fat.

No collections or pneumoperitoneum.

No changes relevant to the clinical context were observed in any of the other structures evaluated.

REPORT B

Appendix showing thickened walls (4 mm) and increased diameter (9 mm). Densification of adjacent adipose planes.

No pneumoperitoneum was observed. No free fluid or collections within the abdomen.

Secondary findings:

- Accessory spleen measuring 1 cm in diameter.

- Bilateral renal cysts, measuring between 0.5 cm and 1.8 cm.

- Small pleural effusion on the right.

No alterations seen in the other structures evaluated.

REPORT C

Liver with normal volume, contours, and density, with no evidence of focal lesions.

Portal and upper hepatic veins unobstructed and of normal caliber.

Biliary tract: no dilatation of the intrahepatic or extrahepatic bile ducts observed.

Spleen of normal volume with homogeneous contrast uptake.

Accessory spleen measuring 1 cm, next to the splenic hilum.

Pancreas of normal morphology and volume, with normal contrast uptake, without signs of ductal dilation or parenchymal calcification.

Adrenal glands with preserved density, morphology, and volume.

Kidneys of normal volume, density, and contours, with preserved parenchymal thickness and symmetric, satisfactory uptake of the contrast medium. Bilateral cortical renal cysts, of regular shape, without septations or calcifications, measuring between 0.5 cm and 1.8 cm.

No signs of nephrolithiasis or dilatation of the renal pelvis or ureter.

Ureters without changes.

Bladder with good filling and normal morphology.

Abdominal aorta and inferior vena cava of normal caliber.

Appendix showing thickened walls (4 mm) and increased diameter (9 mm). Densification of adjacent adipose planes.

No pneumoperitoneum.

No lymph node enlargement or free fluid within the abdominal cavity.

Small pleural effusion on the right.

Conclusion: Acute retrocecal appendicitis with no signs of complication.

REPORT D

**Appendix**

Location: retrocecal

Diameter: 9 mm

Wall thickness: 4 mm

Densification of pericecal adipose planes: present

Pericecal fluid: absent

Pericecal abscess: absent

Periappendicular loop thickening: absent

Appendicolith: present

**Other findings**

Mesenteric lymphadenopathy: absent

Enteric and colonic loops: normal caliber and no wall thickening

**Liver:** no alterations seen

**Gallbladder and biliary tree:** No calcifications. Wall of normal thickness. Absence of intrahepatic and extrahepatic bile duct dilatation.

**Pancreas:** no alterations seen

**Spleen:** no alterations seen

**Adrenal glands:** no alterations seen

**Kidneys and ureters:** simple renal cysts, measuring between 0.5 cm and 1.8 cm, bilaterally.

**Abdominal aorta and main branches:** no alterations seen

**Lung bases:** small pleural effusion on the right

**Organs of the reproductive system:** no alterations seen

**Bones:** no alterations seen

**Conclusion:** Acute retrocecal appendicitis without signs of complication.
